# Supplementary material for: Towards establishing extracellular vesicle-associated RNAs as biomarkers for HER2+ breast cancer
Source: F1000Res. 2021 May 4;9:1362. Originally published 2020 Nov 23. [Version 3] doi: 10.12688/f1000research.27393.3 (PMC7780337; doi:10.12688/f1000research.27393.3)
Supplement: Peer review history

# Peer review: Reviewer response for version 3

**DOI:** 10.5256/f1000research.56245.r84381

**Published:** 2021-05-04

**Authors:** Bruno M. Simoes

**Affiliations:** 1 Manchester Breast Centre, Division of Cancer Sciences, University of Manchester, Manchester, UK

No further comments.

Is the work clearly and accurately presented and does it cite the current literature?

Partly

If applicable, is the statistical analysis and its interpretation appropriate?

Partly

Are all the source data underlying the results available to ensure full reproducibility?

Yes

Is the study design appropriate and is the work technically sound?

Yes

Are the conclusions drawn adequately supported by the results?

Partly

Are sufficient details of methods and analysis provided to allow replication by others?

Yes

Reviewer Expertise:

NA

I confirm that I have read this submission and believe that I have an appropriate level of expertise to confirm that it is of an acceptable scientific standard.

# Peer review: Reviewer response for version 2

**DOI:** 10.5256/f1000research.49630.r79670

**Published:** 2021-02-24

**Authors:** Bruno M. Simoes

**Affiliations:** 1 Manchester Breast Centre, Division of Cancer Sciences, University of Manchester, Manchester, UK

I don't have any further comments. Authors have addressed some of my comments so I'm happy to approve it.

Is the work clearly and accurately presented and does it cite the current literature?

Partly

If applicable, is the statistical analysis and its interpretation appropriate?

Partly

Are all the source data underlying the results available to ensure full reproducibility?

Yes

Is the study design appropriate and is the work technically sound?

Yes

Are the conclusions drawn adequately supported by the results?

Partly

Are sufficient details of methods and analysis provided to allow replication by others?

Yes

Reviewer Expertise:

NA

I confirm that I have read this submission and believe that I have an appropriate level of expertise to confirm that it is of an acceptable scientific standard.

# Peer review: Reviewer response for version 2

**DOI:** 10.5256/f1000research.49630.r79669

**Published:** 2021-02-23

**Authors:** Nagarajan Kannan

**Affiliations:** 1 Division of Experimental Pathology, Department of Laboratory Medicine and Pathology, Center for Regenerative Medicine, Mayo Clinic, Rochester, MN, USA

No further comments.

Is the work clearly and accurately presented and does it cite the current literature?

Yes

If applicable, is the statistical analysis and its interpretation appropriate?

Yes

Are all the source data underlying the results available to ensure full reproducibility?

Yes

Is the study design appropriate and is the work technically sound?

Yes

Are the conclusions drawn adequately supported by the results?

Yes

Are sufficient details of methods and analysis provided to allow replication by others?

Yes

Reviewer Expertise:

Breast biobanking, Mammary gland development, Breast cancer prevention

I confirm that I have read this submission and believe that I have an appropriate level of expertise to confirm that it is of an acceptable scientific standard.

# Peer review: Reviewer response for version 1

**DOI:** 10.5256/f1000research.30272.r75232

**Published:** 2020-12-21

**Authors:** Bruno M. Simoes

**Affiliations:** 1 Manchester Breast Centre, Division of Cancer Sciences, University of Manchester, Manchester, UK

The authors report a novel culture model to isolate and increase the yield of extracellular vesicles (EVs) using a breast cancer cell line. They also claim that some RNA molecules present in EVs are predominantly expressed in tumour tissues compared to their normal tissues’ counterparts.

 My major concerns are the following:

 In Figure 1 – Experiments comparing the results with conventional 2D culture system should be shown to support the statements in the paper. And comparison using bovine serum should be shown too.

 Use of specific EV markers is needed to confirm the presence of EVs – see Gonzalez et al, Plos One, 2014 ^1^.

 Use of at least another cell line is warranted to validate the data and use of primary cells would be ideal to assess potential clinical translation of the data.

 Figure 2A – The rational for selection of this list of EV-associated RNAs is not clear and needs to be defined. Real-time PCR data should be normalized to a reference transcript.

 Also, it is not clear if these experiments were done with large EVs, small EVs or both? If data corresponds only to small EVs then the experiments should be repeated with large EVs too.

 Authors state “RNAs BIRC5 and HOTAIR are promising EV-biomarkers” but this is not proven by the data. The housekeeping genes GAPDH and HPRT are also expressed in EVs. Testing the presence of BIRC5 and HOTAIR RNAs in tumour vs non-tumour EVs is warranted.

 Number of independent experiments and replicates needs to be stated throughout the manuscript.

Is the work clearly and accurately presented and does it cite the current literature?

Partly

If applicable, is the statistical analysis and its interpretation appropriate?

Partly

Are all the source data underlying the results available to ensure full reproducibility?

Yes

Is the study design appropriate and is the work technically sound?

Yes

Are the conclusions drawn adequately supported by the results?

Partly

Are sufficient details of methods and analysis provided to allow replication by others?

Yes

Reviewer Expertise:

Breast cancer biology

I confirm that I have read this submission and believe that I have an appropriate level of expertise to confirm that it is of an acceptable scientific standard, however I have significant reservations, as outlined above.

## References

[1] : Human mammospheres secrete hormone-regulated active extracellular vesicles. *PLoS One*.2014;9(1) : 10.1371/journal.pone.0083955 e83955 10.1371/journal.pone.0083955 24404144PMC3880284

# Peer review: Reviewer response for version 1

**DOI:** 10.5256/f1000research.30272.r75229

**Published:** 2020-12-07

**Authors:** Tracy K. Hale

**Affiliations:** 1 School of Fundamental Sciences, Massey University, Palmerston North, New Zealand

The identification of extracellular vesicle (EV) biomakers is certainly of interest in breast cancer research. This paper presents methodology to grow and isolate sufficient EVs to enable the investigation of their cargo. This study describes the growth of the breast cancer BT474 cell line in a CELLine AD 1000 bioreactor flask with media containing CDM-HD serum. This allowed the cells to be grown in 3D conditions and large numbers of EVs to be isolated without contamination from bovine EVs.

1. Figure 1 could be improved by enlarging region of Fig 1A focusing on the part of the chamber growing the cells and the isolation of EVs. 
2. Could an antibody directed against a histone or tubulin be used in the western blot (Fig 1F) to show that the isolated EVs are not contaminated with any cellular debris?
3. In Figure 2A are the differences in HOTAIR (and the others) significant between BT474 cells and EVs? To what p-value?

The growth of cells in this system shows that there are differential levels of HOTAIR in the cells vs EVs, and gene expression data is presented to demonstrate the over expression of HOTAIR in tumours.

 It will be of interest to test the presence of HOTAIR in malignant vs non-malignant EVs, to support the HOTAIR as a breast cancer EV biomarker.

 The authors discuss the advantages of RNA biomarkers and while a candidate approach is taken here, this ability to isolate sufficient EVs allows them to be used for RNAseq etc.

Is the work clearly and accurately presented and does it cite the current literature?

Yes

If applicable, is the statistical analysis and its interpretation appropriate?

Partly

Are all the source data underlying the results available to ensure full reproducibility?

Yes

Is the study design appropriate and is the work technically sound?

Yes

Are the conclusions drawn adequately supported by the results?

Yes

Are sufficient details of methods and analysis provided to allow replication by others?

Yes

Reviewer Expertise:

Cancer and chromatin biology

I confirm that I have read this submission and believe that I have an appropriate level of expertise to confirm that it is of an acceptable scientific standard.

# Peer review: Reviewer response for version 1

**DOI:** 10.5256/f1000research.30272.r75230

**Published:** 2020-12-07

**Authors:** Nagarajan Kannan

**Affiliations:** 1 Division of Experimental Pathology, Department of Laboratory Medicine and Pathology, Center for Regenerative Medicine, Mayo Clinic, Rochester, MN, USA

Extracellular vesicles (EV) are fast emerging as both therapeutic agents and biomarkers. Low yields of EVs in commonly used experimental models have somewhat diminished interest and their further scrutiny. Methodologies to improve EV yield in short term cultures are desirable and much needed for this field.

 In this brief communication, the authors report a culture method to enrich EV from low EV yielding breast cancer cell line, and using public datasets of tissues to bioinformatically demonstrate that these EVs are associated with RNA species enriched in tumors compared to their respective normal tissues.

 The EV enrichment method appears to be simple and straightforward, and therefore the applications in cancer field is apparent. I have the following comments for the authors.

1. Please provide the rate of EV production using standard 2D culture method vs your novel bioreactor-based method. It may perhaps appeal to labs working in this area to know the rate of production of EV per ml media per 1million seeded cells per hour in culture (both standard vs bioreactor). 
2. Figures: It is not clear how many times these experiments were repeated or number of samples used. Please state them in the Figure Legends. Figure 1C-D showing hydrodynamic diameter distribution profiles should have intervals in Y-axis.
3. The authors should discuss possible drawbacks to this method or choice of cell lines used in this study. 
4. Could you further elaborate on how the TCGA data was analyzed in your methods in order to help reproduce your findings by others.

Is the work clearly and accurately presented and does it cite the current literature?

Yes

If applicable, is the statistical analysis and its interpretation appropriate?

Yes

Are all the source data underlying the results available to ensure full reproducibility?

Yes

Is the study design appropriate and is the work technically sound?

Yes

Are the conclusions drawn adequately supported by the results?

Yes

Are sufficient details of methods and analysis provided to allow replication by others?

Yes

Reviewer Expertise:

Breast biobanking, Mammary gland development, Breast cancer prevention

I confirm that I have read this submission and believe that I have an appropriate level of expertise to confirm that it is of an acceptable scientific standard, however I have significant reservations, as outlined above.
